# Synthesis of multi-walled carbon nanotube/polyhedral oligomeric silsesquioxane nanohybrid by utilizing click chemistry

**DOI:** 10.1186/1556-276X-6-122

**Published:** 2011-02-08

**Authors:** Santosh Kumar Yadav, Sibdas Singha Mahapatra, Hye Jin Yoo, Jae Whan Cho

**Affiliations:** 1 Department of Textile Engineering, Konkuk University, Seoul 143-701, Korea; 2 Department of Chemical Engineering and Chemical Technology, Imperial College, London SW7 2AZ, UK

## Abstract

A new hybrid material consisting of a polyhedral oligomeric silsesquioxane (POSS) and carbon nanotube (CNT) was synthesized by a simple and versatile approach entailing click coupling between azide moiety-functionalized POSS and alkyne-functionalized multi-walled CNTs. This approach provides a simple and convenient route to efficiently functionalize a wide variety of nanoscale nanostructure materials on the surface of CNTs.

## Introduction

A hybrid nanomaterial can be broadly depicted as a multi-component system where two or more nanomaterials are unified to form a new nanomaterial fabricated with the aim of realizing attractive multi-functional properties. Hybrid nanomaterials of carbon nanotubes (CNTs) with metals, metal oxides, and biological compounds have been developed for various applications such as sensors, actuators, solar cells, biosensors, and light emitting devices [1,2]. CNTs offer diverse optical, electrical, and mechanical properties [3,4], making them attractive building blocks for realizing novel functionality via hybridization [5,6].

Polyhedral oligomeric silsesquioxane (POSS), a type of inorganic nanostructured molecule [7-9], contains Si-O cores that have a special cage structure and good solubility. Surrounded by various organic groups, POSS is a strong candidate for further functionalization to develop nanohybrid materials [10-12]. The functionalization of CNTs has been one of the most intensively explored methods to produce CNT-based nanostructure materials. Various functionalization strategies for CNTs can be performed with non-covalent bonding, such as van der Waals and π-π interaction, as well as by covalent bonding, such as acid treatment, oxidation, esterification, amidation, radial coupling, anionic coupling, and click coupling [13,14]. These functionalization methods are dependent on the type, distribution, and concentration of compounds, i.e., polymers, metals, or inorganic nanoparticles, on the surface of the CNTs [15]. Since a landmark report by Sharpless and co-authors [16], Cu(I)-catalyzed [3+2] Huisgen cycloaddition reaction of azides and alkynes moieties, referred to as "click chemistry," has received a great deal of attention from researchers in fields ranging from organic synthesis to materials chemistry.

This article describes the synthesis of a CNT-POSS nanohybrid material using a click chemistry reaction. It is anticipated that this approach can be utilized to prepare nanohybrids with high interfacial bonding.

## Experimental

### Materials

Multi-walled carbon nanotubes (MWNTs) used in this study were purchased from Iljin Nano Tech, Seoul, Korea. Their diameter and length ranges were approximately 10-20 nm and 20 μm, respectively. EP0402-epoxycyclohexyllsobutyl POSS (Hybrid Plastic Co. Hattiesburg, MS, USA), propargyl bromide, *p*-nitrophenol, terabutylammonium bromide, 3-methyl butyl nitrite, copper iodide, and 1,8-diazabicyclo[5,4]undecene-7-ene were used without further purification.

### Characterization

Fourier transform-infrared (FT-IR) spectroscopic measurements were performed using a Jasco FT-IR 300E device. Elemental analysis was determined by Perkin-Elmer analyzer model 2400 CHN analyzer. ^1^H NMR and ^13^C NMR spectra were measured on a 400-MHz instrument by Bruker on CDCl_3 _solutions at room temperature. Raman spectroscopy (LabRam HR Ar-ion laser 514 nm, Jobin-Yvon, Longjumeau, France) was used to confirm the functionalization of MWNTs. X-ray photoelectron spectroscopy (XPS, ESCSA 2000) was used to analyze the surface composition of the nanotubes. Observation of the surface morphology and energy dispersive X-ray spectrum (EDX) measurement of the MWNT-POSS nanohybrid was carried out by transmission electron microscopy (TEM, JEM 2100F, JEOL). Thermogravimetric analysis (TGA) was carried out in a TA Q 50 system TGA.

### Preparation of alkyne-functionalized MWNTs

For the click reaction, *p*-aminophenyl propargyl ether was first synthesized according to a procedure reported in the literature [17] to introduce alkyne-functionality on the CNTs. Initially, 60 mg of MWNTs and 3.0 g of *p*-aminophenyl propargyl ether were placed in a two-necked flask fitted with a reflux condenser and a magnetic stirrer bar under a N_2 _atmosphere. Then, 3.0 g 3-methyl butyl nitrite was slowly injected via a syringe, and the reaction mixture was stirred at 60°C for 5 h. The resulting product was washed three times with 100 ml of dimethylformamide (DMF), and dried under vacuum at 60°C for 80 h, and the product yield was 80%.

### Azidation of POSS molecules

**Figure 1:**
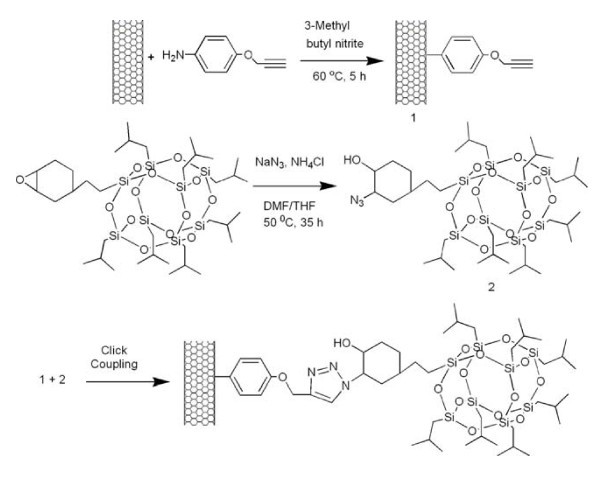
**Strategy for "clicking" POSS molecule onto MWNTs**.

The azidation of the POSS molecule was carried out with sodium azide in the presence of ammonium chloride, as shown in Figure 1. Typically, a solution of POSS (1.0 g 3.19 mmol) in tetrahydrofuran (THF) (5 ml) was added to a solution of sodium azide (208 mg 3.19 mmol) and ammonium chloride (170 mg 3.18 mmol) in DMF (5 ml), and the mixture was stirred for 35 h at 50°C. The mixture was precipitated into 200 ml of water and the product was vacuum dried at 40°C for 60 h. The yield of azide-functionalized POSS obtained was 85%.

### Synthesis of MWNT-POSS nanohybrid by click coupling

Coupling of an azide moiety-containing POSS and alkyne-functionalized MWNTs was carried out via Cu(I)-catalyzed click chemistry. Typically, 20 mg of alkyne-functionalized MWNTs was dispersed in 15 ml of DMF. The MWNTs solution was added to a two-necked flask containing a 400 mg (0.43 mmol) solution of POSS-N_3 _in 15 ml of DMF. The flask was equipped with a magnetic stirrer bar with a reflux condenser. 162 mg (0.85 mmol) of copper iodide and 6.4 g (42.5 mmol) of 1,8-diazabicyclo[5,4]undecene-7-ene were charged to the above homogenous solution, which was then heated at 60°C with continuous stirring for 24 h under a nitrogen atmosphere. The product was precipitated into 200 ml of water followed by 100 ml of THF for three times to remove unreacted POSS molecules. The product was dried overnight under vacuum at room temperature, and the product yield was 75-80%.

## Result and discussion

**Figure 2:**
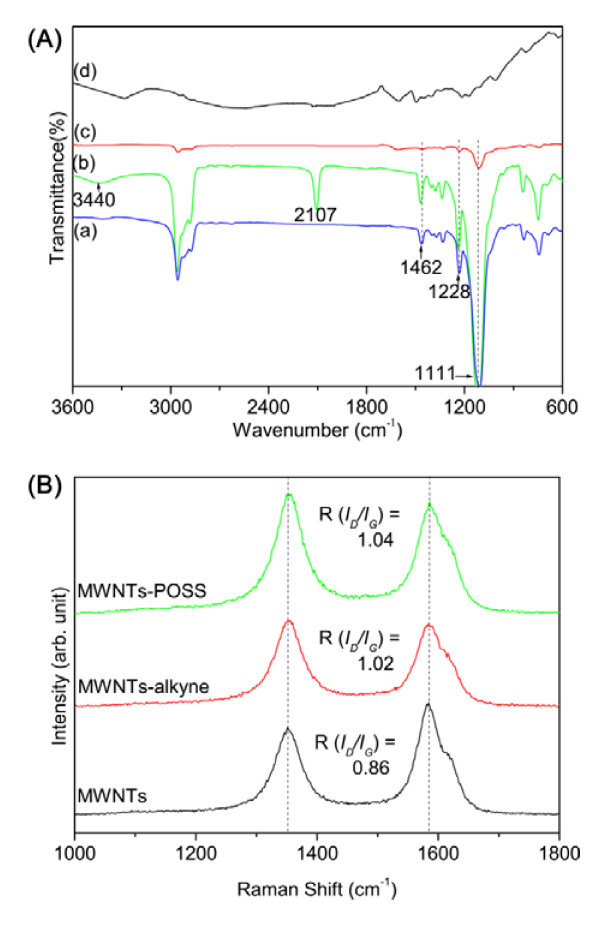
**FT-IR and Raman spectra of nanomaterials**. **(A) **IR spectra of pure POSS (a), POSS-N_3 _(b), MWNT-POSS nanohybrid (c), and MWNTs-alkyne (d). **(B) **Raman spectra of pristine MWNTs, MWNTs-alkyne, and MWNT-POSS nanohybrid.

**Figure 3:**
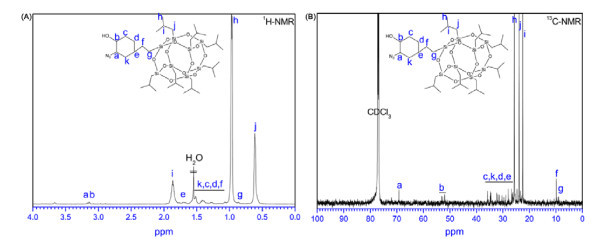
**^1^H NMR and ^13^C NMR spectra of azide functionalized POSS**. **(a) **^1^H NMR spectrum of POSS-N_3 _and **(b) **^13^C NMR spectrum of POSS-N_3_.

The aim of this study is to prepare covalently functionalized MWNT-POSS nanohybrids by click chemistry between azide-functionalized POSS (POSS-N_3_) and alkyne-functionalized MWNTs (Figure 1). Alkyne-functionalized MWNTs are prepared via a solvent-free diazotization reaction and a coupling reaction between MWNTs and *p*-aminophenyl propargyl ether. POSS-N_3 _is prepared by a simple reaction of POSS with sodium azide in the presence of ammonium chloride. The success of click cycloaddition is supported by evidence from FT-IR, Raman, XPS, TEM, EDX, and TGA. As a confirmation of the reactions, Figure 2a shows the IR spectra of pure POSS, which has characteristic peaks at 1111 cm^-1 ^for Si-O-Si stretching [18], 1462 cm^-1 ^for CH_2 _stretching of cyclohexyl [19], and 1228 cm^-1 ^for Si-CH_2 _stretching [20]. The azidation of the POSS molecule was also confirmed by comparison of the IR spectrum of pure POSS with that of POSS (POSS-N_3_) with azide-functionality. A new peak at 2107 cm^-1 ^corresponding to the azide group [21], and simultaneously another peak at 3440 cm^-1 ^for OH stretching were observed. The results of ^1^H NMR and ^13^C NMR measurements reveal clearly the POSS-N_3 _structure (Figure 3a,b). The charecteristic signals at δ = 3.18 and 3.12 ppm in ^1^H NMR, and δ = 69.2 and 52-53 in ^13^C NMR are assigned to the -CH proton and carbon of cyclohexane combined with -OH and N_3 _groups, respectively. Elemental anlysis results are also in good agreement with experimental values (Table 1), confirming the successful azidation of POSS. The click coupling between the alkyne-functionalized MWNTs and azide-functionalized POSS in the presence of Cu(I) catalyst provided a 1,2,3-triazole ring. This indicates that the POSS molecule is successfully attached to the surface of the MWNTs. Thus, the IR spectra of MWNT-POSS nanohybrid, featuring a azide peak of POSS molecules at 2107 cm,^-1 ^completely disappeared, indicating the formation of 1,2,3-triazole after the click reaction.

**Table 1:** Elemental analysis data of POSS-N_3_

**POSS-N**_**3**_	C%	H%	N%
Calculated	43.90	7.88	4.26
Found	44.26	7.65	4.01

**Figure 4:**
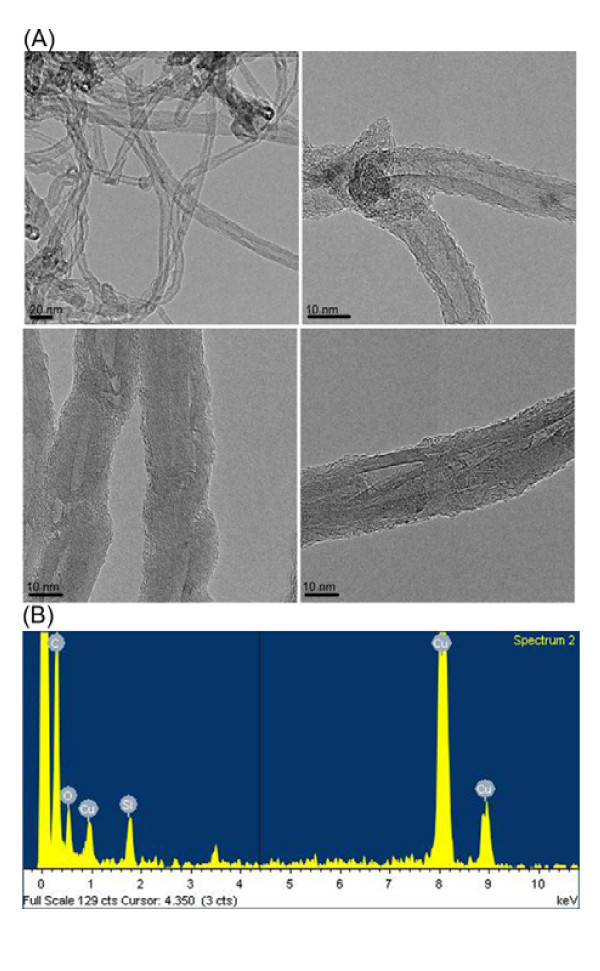
**TEM images and EDX spectra of nanohybrid**. **(a) **TEM images of MWNT-POSS nanohybrid and **(b) **EDX spectra of MWNT-POSS nanohybrid.

Raman spectroscopy can be used as a powerful tool for characterizing functionalized CNTs. Figure 2b shows that the pristine MWNTs, MWNTs-alkyne, and the MWNT-POSS nanohybrid have two characteristic bands at 1352 cm^-1 ^(D band) and 1585 cm^-1 ^(G band) [22]. The D band is attributed to a disordered graphite structure or *sp*^3^-hybridized carbons of the nanotubes, whereas the G band corresponds to a splitting of the E_2_g stretching mode of graphite, which reflects the structural intensity of the *sp*^2^-hybridized carbon atoms. The increase in the band intensity ratio (*I*_D_/*I*_G_) of the functionalized MWNTs reflects the relative degree of functionalization or defects in the nanotubes, indicating covalent functionalization MWNT-POSS nanohybrids. TEM images of the MWNT-POSS nanohybrid (Figure 4a) show that MWNTs are grafted by the POSS molecules. This shows strong evidence that the POSS molecules are well coated on the surface of the MWNTs. These results are also strongly supported by the EDX with copper as a substrate (Figure 4b).

**Figure 5:**
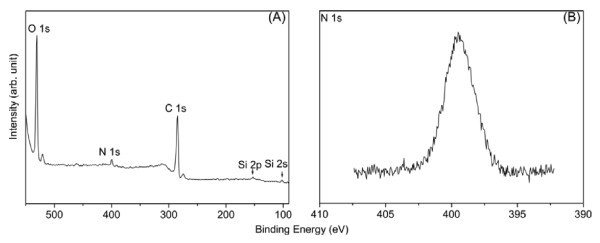
**XPS spectra of MWNT-POSS nanohybrid**. **(a) **Wide scan spectra of MWNT-POSS nanohybrid and **(b) **N (1s) high-resolution peak for MWNT-POSSS nanohybrid.

**Figure 6:**
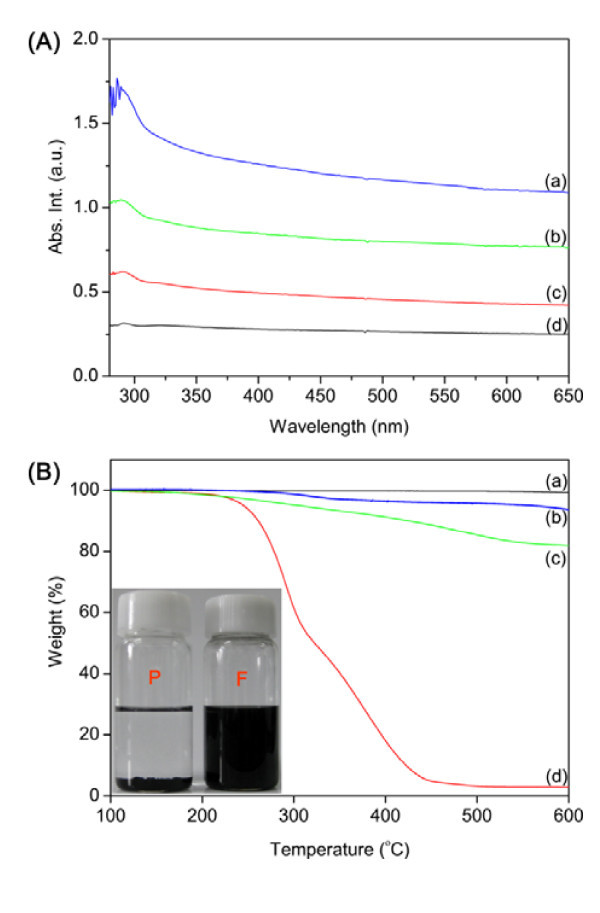
**UV-Vis absorption spectra and TGA analysis of nanomaterials**. **(A) **UV-Vis absorption spectra of MWNT-POSS nanohybrid in different concentrations: (a) 0.01 mg/ml, (b) 0.005 mg/ml, (c) 0.002 mg/ml, and 0.001 mg/ml in THF. **(B) **TGA analysis of pristine MWNTs (a), MWNT-POSS nanohybrid (b), pure POSS (c), solubility test results (inset) of pristine MWNTs (P), and MWNT-POSS nanohybrid (F).

Furthermore, XPS was additionally used to investigate the clicked surface. The XPS spectra of MWNT-POSS nanohybrid material are shown in Figure 5A. Three characteristic peaks at 285, 532, and 400 eV were observed for C 1s, O 1s, and N 1s, respectively. Two relatively weak signals were also observed at 102 and 152 eV, which are characteristic peaks of Si 2s and Si 2p, respectively, from the POSS cage. The N (1s) high-resolution peak for the MWNT-POSS nanohybrid (Figure 5B) suggests the presence of only one oxidation state of the nitrogen atom due to the formation of a 1,2,3-triazole ring [23], which confirms that the POSS-N_3 _molecule reacted with alkyne-functionalized MWNTs. The atomic percent and weight percent of Si for the MWNT-POSS nanohybrid were calculated by EDX measurment as 3.98 and 8.57%, respectively (Table 2). These results indicate the presence of POSS molecules on the surface of the MWNTs. The MWNT-POSS nanohybrid showed a typical electronic absorption spectrum of solubilized CNTs, and the absorbance decreased gradually in the UV to visible region (Figure 6a). As the POSS molecules have better reactivity and solubility in organic solvent, functionalization of POSS molecule with CNTs can substantially enhance the solubility and processability of the nanohybrid. Figure 6b (inset) shows the solubility test results of pristine MWNTs and the MWNT-POSS nanohybrid in THF at a concentration of 2.5 mg/mL. It is observed that the MWNT-POSS nanohybrid shows better dispersion stability than pristine MWNTs in THF after 4 weeks. The TGA analysis provides further evidence for functionalization of MWNTs with POSS (Figure 6b). TGA results show weight losses of 2, 6, and 19% at 700°C for pristine MWNTs, alkyne-functionalized MWNTs, and the MWNT-POSS nanohybrid, respectively. The difference in weight loss of alkyne-functionalized MWNTs and the MWNT-POSS nanohybrid is attributed to the presence of POSS molecules on the surface of the MWNTs [24,25]. TGA data of POSS show almost complete mass loss at temperatures over 450°C due to its sublimation [10].

**Table 2:** Atomic % and weight % of MWNT-POSS nanohybrid determined from EDX experimental data

Element	Weight %	Atomic %
C	79.31	86.14
O	12.11	9.88
Si	8.57	3.98

## Conclusion

In summary, the synthesis of a MWNT-POSS nanohybrid was accomplished via Cu(I)-catalyzed azide-alkyne cycloaddition between azide moiety-containing POSS and alkyne-functionalized MWNTs. Click coupling can provide a new strategy for the synthesis of CNT-based nanohybrids.

## Abbreviations

CNT: carbon nanotube; DMF: dimethylformamide; EDX: energy dispersive X-ray spectrum; FT-IR: Fourier transform infrared; MWNTs: multi-walled carbon nanotubes; POSS: polyhedral oligomeric silsesquioxane; TEM: transmission electron microscopy; TGA: thermogravimetric analysis; THF: tetrahydrofuran; XPS: X-ray photoelectron spectroscopy.

## Competing interests

The authors declare that they have no competing interests.

## Authors' contributions

SKY conducted all the experiments and drafted the manuscript. SSM helped in technical support for experiments and characterization. HJY participated in measurements and data analysis. JWC designed the experiments and supervised the all of the study. All the authors discussed the results and approved the final manuscript.
